# NutriDiary, a Smartphone-Based Dietary Record App: Description and Usability Evaluation

**DOI:** 10.2196/62776

**Published:** 2025-02-10

**Authors:** Linda Klasen, Stefanie Anna Julia Koch, Maike Elena Benz, Johanna Conrad, Ute Alexy, Konrad Blaszkiewicz, Ionut Andone, Ute Nöthlings

**Affiliations:** 1 Nutritional Epidemiology, Institute of Nutrition and Food Sciences, University of Bonn, Friedrich-Hirzebruch-Allee 7, Bonn, 53115, Germany, 49 228 73 6049; 2 German Nutrition Society (DGE), Bonn, Germany; 3 Department of Computer Science, University of Bonn, Bonn, Germany

**Keywords:** dietary assessment, food record, barcode scanning, app, mobile phone

## Abstract

**Background:**

Repeated applications of short-term dietary assessment instruments are recommended for estimating usual dietary intake. For this purpose, NutriDiary, a smartphone app for collecting weighed dietary records (WDRs) in the German population, was developed.

**Objective:**

We aim to describe NutriDiary and evaluate its usability and acceptability.

**Methods:**

NutriDiary was developed as a WDR, allowing users to enter food items via text search, barcode scanning, or free text entry. The sample for the evaluation study included 74 participants (n=51, 69% female, aged 18‐64 years), including 27 (37.5%) experts and 47 (63.5%) laypersons (including n=22, 30%, nutrition students). Participants completed a 1-day WDR and entered a predefined sample meal (n=17 foods) the following day by using NutriDiary. An evaluation questionnaire was answered from which the system usability scale (SUS) score (0‐100) was calculated. A backward selection procedure (PROC REG in SAS; SAS Institute) was used to identify potential predictors for the SUS score (age, sex, status [expert or laypersons], and operating system [iOS or Android]).

**Results:**

The median SUS score of 75 (IQR 63‐88) indicated good usability. Age was the only characteristic identified as a potential predictor for a lower SUS score (*P*<.001). The median completion time for an individual WDR was 35 (IQR 19‐52) minutes. Older participants took longer to enter the data than younger ones (18‐30 y: median 1.5, IQR 1.1‐2.0 min/item vs 45‐64 y: median 1.8, IQR 1.3‐2.3 min/item). Most participants expressed a preference for NutriDiary over the traditional paper-based method.

**Conclusions:**

Good usability and acceptability make NutriDiary promising for use in epidemiological studies.

## Introduction

Usual dietary intake, the long-term average daily intake of a nutrient or food**,** is the relevant exposure when studying diet-health relationships in nutritional epidemiology. To estimate usual dietary intake, repeated applications of short-term dietary assessment instruments are recommended [1]. Self-reported dietary intake is the most commonly used method in large-scale studies through its rapid and cost-effective use. Mostly, instruments such as 24-hour dietary recalls, food frequency questionnaires or dietary records (DRs) are used, with each of these methods having individual limitations and strengths [2]. Repeated use of these traditional dietary assessment instruments is costly and burdensome for both participants and researchers [3,4]. Innovative technologies such as web- or smartphone-based tools have the potential to facilitate self-reported dietary assessment by reducing the costs and time effort of data collection and postprocessing while achieving higher acceptance in study participants [5-7]. Smartphone technology seems particularly promising among those innovative approaches. First, smartphones are widely available to a large proportion of the population. According to a survey conducted in 2021, over 95% of people aged older than 13 years in Germany already use a smartphone [8]. Second, due to the advantage of portability and the fact that most people always carry their smartphone with them, smartphone-based tools are well suited for real-time recording of food intake [1]. Third, smartphone apps can facilitate food entry through the supplementary use of an integrated barcode scanner or by using the camera function, which reduces the need to look up every food item in a database or enter it manually [9]. Nowadays, a large number of commercial nutrition apps for self-tracking or nutritional advice are freely available [10,11]. However, because of their limited scope and questionable quality of nutrient information, those apps tend to be unsuitable for dietary assessment in epidemiological studies [12,13]. Thus, several DR apps have been developed specifically for use in epidemiological studies. In a systematic review by König et al [14], 5 core assessment features to collect data on dietary intake in scientific studies by smartphone apps were identified: photo-based assessment, assessment of serving or portion sizes, free-text description of food intake, selection from a food database, and classification systems. These features are used either alone or in combination. Thereby, the combination of photo-based recording with free-text descriptions of the consumed foods or the joint use of a food database and the assessment of serving or portion sizes were the most popular methods [14].

To provide a digital alternative for paper-based dietary assessment in epidemiological studies, we developed NutriDiary, an app for conducting weighed dietary records (WDRs) with an integrated barcode scanner. This paper aims to describe the current version of NutriDiary and to report on its usability and acceptability in laypersons and experts.

## Methods

### NutriDiary and the NutriDiary Database

#### Development and Functions of NutriDiary

**Figure 1.**
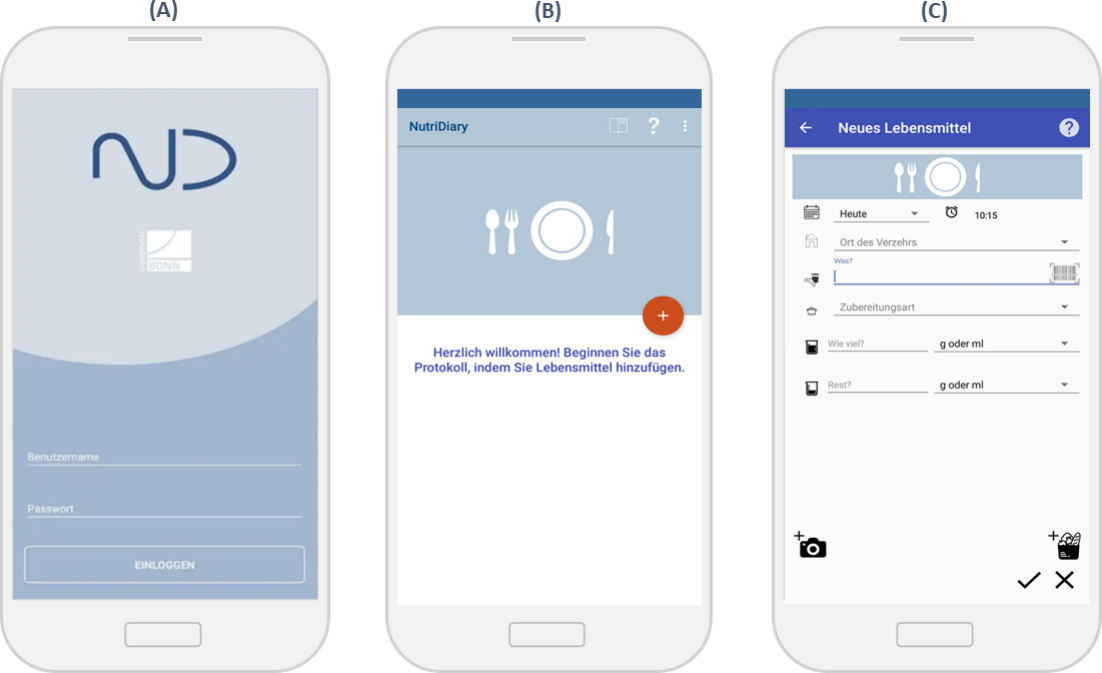
The NutriDiary app (android version): (A) screen for entering access data, (B) welcome screen, and (C) food entry.

NutriDiary was developed as a smartphone app to conduct WDRs within nutritional epidemiological studies. The app is available in common app stores and study participants (hereafter referred to as users) can use the app on their smartphones with personal login data (Figure 1A). Users can start their WDR after agreeing to the data protection regulations (Figure 1B). When entering a new eating occasion, users are initially asked to enter the date, time, and place of consumption (Figure 1C). Then, the app offers three ways to enter food, beverages, and supplements: (1) a text search and subsequent selection from the underlying database, (2) barcode scanning in the underlying database, or (3) free text entry.

**Figure 2.**
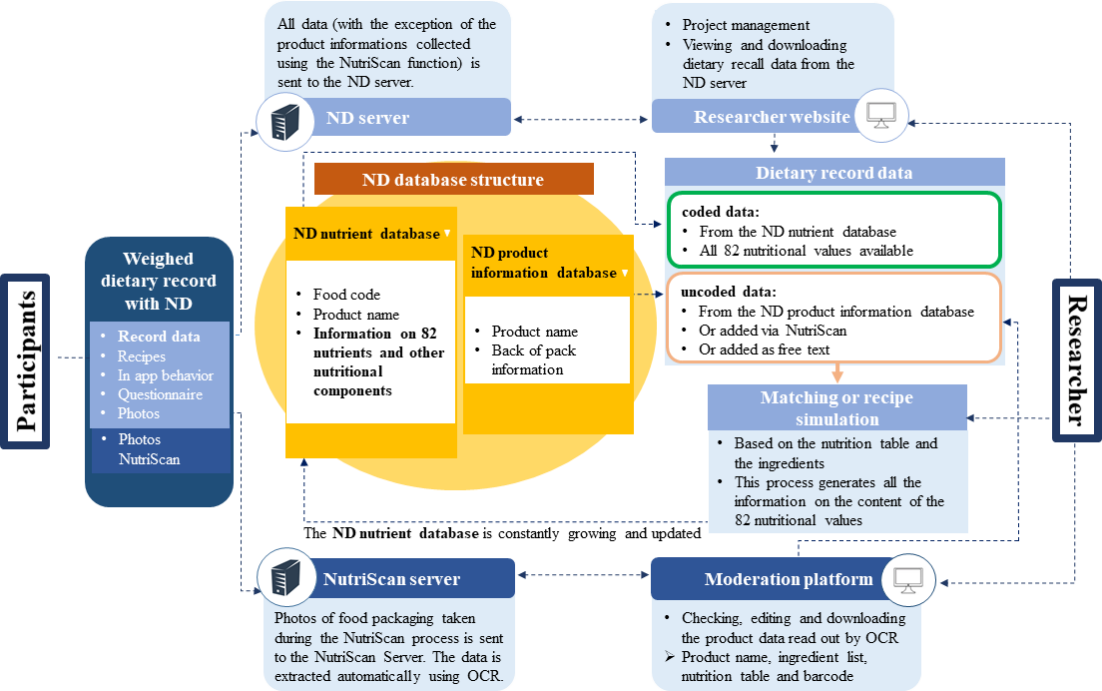
Overview of the data structure and data flow of the NutriDiary app; ND: NutriDiary; OCR: optical character reading.

If a food item cannot be identified via barcode scanning, the user is guided through a standardized process for collecting all relevant product information (hereinafter referred to as the NutriScan process). Thereby, the user is asked to take photos of the brand and product name, the barcode, the ingredient list and the nutrient table following step-by-step instructions (Multimedia Appendix 1). This data is then sent to the NutriScan server and automatically read out using optical character reading. Researchers can access, edit, and download this data via a moderation platform. Based on the packaging information, dieticians can match detailed nutrient data from a similar product in the database or estimate detailed nutrient values by recipe simulation in order to continuously update and expand the underlying database (Figure 2). The recipe simulation is carried out manually using the list of ingredients and the nutritional table and is described in detail elsewhere [15]. Finally, users enter the weighed amount consumed, preparation method, and quantity of potential leftovers (Figure 1C). If weighing is not possible, users are provided with a range of specified options in the drop-down menu to select an estimated portion size, such as teaspoon or slice. After all foods and beverages have been entered, users are redirected to the main screen, where all entered eating occasions are displayed for final review. To further ease the process of recording, NutriDiary offers some usability features. A recipe editor allows entry of custom recipes, which will be added to the user’s personal databases. An integrated help mode provides immediate and problem-specific assistance to users by simply touching the screen element they need help with. In addition, the app includes a photo function for collecting information, for example, on meals consumed out-of-home and entered via free text. After finishing the WDR, data are submitted to a server of the University of Bonn (NutriDiary server, Figure 2). This server also provides an administration tool to researchers (researcher website) where scientific personnel can select project-specific settings, for example, study name, study duration or the number of recording days. The app also offers the option to integrate an individual questionnaire at the end of the recording period.

#### Description of the NutriDiary Database

The complex database structure of NutriDiary is shown in Figure 2. It currently contains more than 150,000 items (approximately 25% are duplicates due to different packaging sizes and different barcodes) which come from various sources. The core database is divided into the NutriDiary nutrient database and the NutriDiary product information database. The basis of the NutriDiary nutrient database is an adaptation of the in-house food and nutrient database of the DONALD (Dortmund Nutritional and Anthropometric Longitudinally Designed) study named LEBTAB (LEBensmittelTABelle, food table) [15-17]. LEBTAB currently contains around 19,000 generic and branded food items (as of June 2023) with corresponding contents of energy, 82 nutrients, and other nutritional components. Data entries for generic foods are based predominantly on the German national standard food database “Bundeslebensmittelschlüssel” (version 3.02 [18]; Federal Ministry of Food and Agriculture). The energy and nutrient contents of branded foods are predominantly estimated by recipe simulation using labeled ingredients and declared nutrient contents [15,17]. When the participant enters a food item from the NutriDiary nutrient database, this item is automatically coded and the corresponding values for energy and 82 nutrients are available. To enhance user-friendliness and allow barcode scanning, additional branded food products and barcode information were added to the NutriDiary database structure by:

- requesting product information (product name, barcode, ingredient list, and nutrition table) from food manufacturers and others (Atrify [19], a subsidiary of GS1 Germany [1WorldSync] and DATA NatuRe eG [20], a cooperative to create a central data pool for organically produced foods) to set up the NutriDiary product information database, and
- adding barcode information of already included branded products to the NutriDiary nutrient database by searching in open databases such as Open Food Facts [21], codecheck [22] (Producto Check GmbH), and Open EAN/GTIN Database (European Article Number [EAN]/Global Trade Item Number [GTIN]) [23].

For the branded products obtained through step 1 (NutriDiary product information database), information only on the nutritional values indicated on the packaging (big 7) and the ingredient list are available (Figure 2). When a participant reports one of these products in a WDR, the trained dieticians match extended nutrient values of an equivalent food from the NutriDiary nutrient database or carry out a recipe simulation as already mentioned above [15]. This step generates all 82 nutrients for these products and they are then transferred to the NutriDiary nutrient database, which is constantly growing.

### The NutriDiary Evaluation Study

#### Study Design

A layperson and expert evaluation was conducted. Participants were asked to keep an individual WDR with NutriDiary for 1 day and to enter a predefined sample meal on the following day. The sample meal was identical for all participants and included 4 meals (breakfast, snack, lunch, and dinner) and was provided as a digital presentation sent to study participants via email. The sample meal comprised both, generic (n=15, presented as text) and branded food items (n=3, presented as pictures of the packaging including barcode), all labeled with a hypothetical quantity of consumption, preparation method, and type of entry (text entry vs barcode scanning). Participants were generally instructed to enter branded products via scanning the barcode. Further, 1 product was intentionally not available in both NutriDiary databases, requiring participants to complete the NutriScan process in order to add the food item to the record. Furthermore, participants were tasked to correct a logged entry and to enter a hypothetical leftover without further instructions but with reference to the NutriDiary website [24] to test whether the aids provided there (frequently asked questions and help videos) fulfill their purpose (ie, helping users to help themselves). After participants completed their WDR and entered the sample meal, they were asked to answer an app-integrated evaluation questionnaire on usability and acceptability of NutriDiary on day 3. As the WDRs were not analyzed at the nutrient level, no scales were handed out for weighing the food on day 1. The participants were asked to use scales from their households.

All participants were provided with a short video giving key instructions on how to use NutriDiary before starting the WDR. The video gave a brief overview of how to use the help mode, enter foods (via text search, barcode scanning, or free text entry) and navigate the recipe book. Furthermore, participants were informed about the NutriDiary website [24], where frequently asked questions and help videos for both, iOS and Android can be found. Beyond this, participants did not undergo any additional training in using the app.

#### Recruitment of This Study’s Population

We aimed to recruit a minimum of 51 participants, based on the assumptions outlined by Lewis and Sauro [25]. This recommendation takes into account an SD of 17.7, which is typical for system usability scale (SUS) scores [25], and ensures sufficient precision to achieve a 95% CI with a margin of error of ±5 points. This study recruited both laypersons and experts. Experts (trained nutritionists with experience in the field of dietary assessment) were recruited via direct invitations (n=28). Laypersons (n=52) were recruited via oral advertisement in lectures, mailing, and personal contact by students studying nutrition and food science at the University of Bonn, and as part of a student project. The latter mainly targeted participants between 30 and 60 years of age (in the personal environment of the students and in 2 gyms) in order to increase the number of participants in middle age and older in the group of laypersons. All participants had to be fluent in the German language, have a functioning smartphone and a valid email address. Written informed consent from all participants was obtained before enrollment.

#### Usability and Acceptability Assessment

The questionnaire on usability and acceptability included 14 questions on 3 different categories (usability, acceptance, and technical issues). The usability of NutriDiary was assessed by using the SUS by Brooke [26,27], which allows for comparison between similar systems or products. In short, the SUS is a Likert scale consisting of 5 positive statements (odd-numbered) and 5 negative statements (even-numbered) to which respondents indicate their degree of agreement on a scale from 1 (strong disagreement) to 5 (strong agreement). For odd items, 1 is subtracted from the user response, and for even-numbered items, 5 is subtracted from the user response, added up and multiplied by 2.5 to convert the score ranging from 0 to 100, whereas higher scores indicate better usability. According to Bangor et al [28], an SUS score below 50 is considered as “not acceptable,” a score between 50‐70 years as “marginal,” and a score above 70 as “acceptable” [28,29]. In our study, an appropriate German translation of the SUS developed by SAP usability professionals of a German software corporation (SAP SE; Systems, Applications & Products in Data Processing Societas Europaea) was used and integrated into the usability questionnaire [30].

Age and sex of the participants were assessed within the evaluation questionnaire. Status (expert or layperson) was already categorized during the recruitment process. Information on the operating system was automatically recorded when NutriDiary was used and sent to the server together with the questionnaire data. To find out whether participants would prefer the app to a traditional paper-based WDR, 2 additional questions in the same structure of the SUS were added. In order to assess technical problems participants were asked whether technical errors occurred (yes or no) and, if yes, to describe the error in more detail (free text entry).

Furthermore, in-app behavior of users was recorded and evaluated. The completion time for a WDR with NutriDiary was determined by using the activity protocol of the app, in which all actions were recorded and time-stamped. For this, the time of all input activities was summed up. Interruptions of more than 5 minutes were counted as breaks and excluded from summation. As the time effort of a WDR depends on the complexity of the meals and the number of foods eaten, the relative completion time (completion time divided by the number of items) was additionally calculated. Furthermore, the percentage of estimated household measurements was of interest as well as the percentage of automatically coded WDR entries.

#### Statistical Analyses

Results and participants’ characteristics are presented as median with their lower IQRs for continuous variables or as relative frequencies (%) for categorical variables. A backward selection procedure (PROC REG in SAS) was used to identify characteristics of participants that were potential predictors for the SUS score. The following variables were tested: sex (male or female), age (years), status (expert or layperson), and operating system (iOS or Android). All statistical analyses were conducted using SAS (version 9.4). The significance level was set at *P*<.05.

### Ethical Considerations

All examinations were carried out with written informed consent from study participants. The NutriDiary evaluation study was approved by the Ethics Committee of the University of Bonn (project identification 445/23). All data collected in this study has been pseudonymized to protect participant privacy. No reimbursement was provided to participants for their involvement in this study.

## Results

From overall 80 study participants, 74 (27 experts) completed the NutriDiary evaluation study according to this study protocol and answered the evaluation questionnaire (Table 1). Most participants were female (51/74, 69%) and younger than 30 years of age (41/74, 55%).

The overall age ranged from 18 to 64 years and the median age was 29 years (IQR 25‐45). Overall, 54% (40/74) of the participants owned a smartphone with an iOS operating system. Table 2 shows the SUS score for NutriDiary for the total study sample and stratified by sex, age groups, status, and operating system. In the overall study sample, the SUS score for NutriDiary ranged from 43 to 100 (data not shown). The median SUS score of 75 (IQR 63‐88) indicates a good usability of NutriDiary. The mean SUS score was 74 (SD 15; 95% CI 70-77; data not shown). The median SUS score was higher in women than in men (80, IQR 65‐88, vs 70, IQR 55‐78) and higher in the group of experts than in the group of laypersons (80, IQR 65‐88 vs 73, IQR 58‐88), with female laypersons having a higher SUS score than female experts (83, IQR 66‐88 vs 80, IQR 60‐85). Looking at the group of laypersons after excluding nutrition students (labeled in Tables1  2 as “others,” median age 55, IQR 29‐58, years), the median SUS score was 63 (IQR 50‐73).

**Table 1.** Characteristics of study participants of the NutriDiary evaluation study (N=74).

	Total	Experts	Laypersons		
			Total	Nutrition students	Others
Participants, n (%)	74 (100)	27 (36.5)	47 (63.5)	22 (47)	25 (53)
**Sex (group), n (%)**					
Women	51 (69)	23 (85)	28 (60)	19 (86)	9 (36)
Age, median (IQR)	29 (25‐45)	35 (28‐40)	27 (23‐56)	23 (22‐26)	55 (29‐58)
**Age (years), n (%)**					
Age group 1: 18-≤30	41 (55.4)	12 (44.4)	29 (61.7)	22 (100)	7 (28)
Age group 2:>30-≤45	15 (20.3)	11 (40.7)	4 (8.5)	0 (0)	4 (16)
Age group 3:>45‐65	18 (24.3)	4 (14.8)	14 (29.8)	0 (0)	14 (56)
**Operating system, n (%)**					
iOS	40 (54)	12 (44.4)	28 (59.6)	12 (54.5)	16 (64)
Android	34 (46)	15 (55.6)	19 (40.4)	10 (45.5)	9 (36)

**Table 2.** System usability scale (SUS) score for the NutriDiary app presented as median (IQR) values.

			Total		Women		Men	
			n	Median (IQR)	n	Median (IQR)	n	Median (IQR)
SUS score			74	75 (63‐88)	51	80 (65‐88)	23	70 (55‐78)
**Stratified by age group**								
	Age group 1: 18 -≤30 years		41	80 (70‐88)	31	83 (70‐88)	10	71 (63‐85)
	Age group 2: >30 -≤45 years		15	80 (68‐90)	11	85 (65‐93)	4	74 (70‐78)
	Age group 3: >45‐65 years		18	58 (50‐70)	9	58 (48‐65)	9	65 (50‐70)
**Stratified by status**								
	Experts		27	80 (65‐88)	23	80 (60‐85)	4	83 (70‐90)
	**Laypersons (total)**		47	73 (58‐88)	28	83 (66‐88)	19	70 (55‐73)
		Nutrition students	22	85 (75‐88)	19	88 (78‐90)	3	73 (70‐85)
		Others	25	63 (50‐73)	9	55 (48‐65)	16	66 (53‐73)
**Stratified by operating system**								
	iOS		40	78 (65‐88)	26	80 (68‐90)	14	66 (55‐73)
	Android		34	74 (58‐85)	25	75 (58‐85)	9	73 (70‐85)

Among the age groups 1 (18-≤30 y) and 2 (>30-≤45 y), the median SUS score was noticeably higher (80, IQR 70‐88 and 80, IQR 68‐90) than in age group 3 (58, IQR 50‐70). The results of the backward selection procedure showed that age was the only characteristic identified as a potential predictor for the SUS score in the examined sample (*P*<.001).

**Figure 3.**
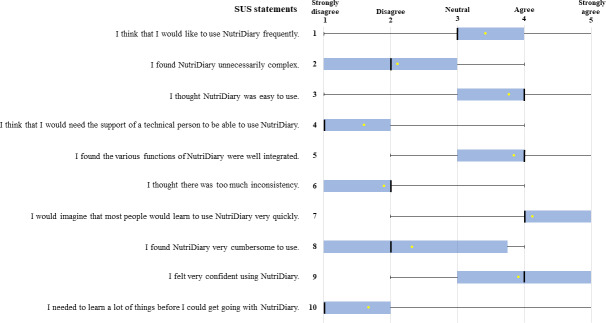
User rating (N=74) of the individual statements of the SUS evaluating the usability and acceptability of NutriDiary, shown as box plots. The dot represents the mean and the thick stripe the median. SUS: system usability scale.

Figure 3 shows the individual statements of the SUS questionnaire and a summary of the answers given by the 74 participants, presented as box plots. The figure shows that agreement tends to be high for the positive (odd-numbered) statements and tends to be low for the negative (even-numbered) statements, as is a prerequisite for a higher SUS score. Among the positive statements, the participants showed the lowest level of agreement with statement 1 “I think that I would like to use NutriDiary frequently.” For the negative statements, agreement was highest for statement 8 “I found NutriDiary very cumbersome to use.”

Table 3 shows the results of the evaluated in-app behavior logs and WDRs. The calculated median completion time for an individual WDR (day 1) with NutriDiary was 35 (IQR 19‐52) minutes. Participants needed a median of 1.6 (IQR 1.2‐2) minutes to enter an item. This relative completion time was slightly higher in age group 3 (>45‐63 y) compared to younger participants (1.8, IQR 1.3‐2.3, min/item vs 1.5, IQR 1.1‐2, min/item). The median time it took participants to enter the sample meal in NutriDiary on day 2 was 15 (IQR 12‐18) minutes. The median relative completion time for entering the sample meal was 0.8 (IQR 0.7‐0.9) min/item. Participants in age group 3 took slightly longer than younger participants. The median proportion of already coded items was 68% (IQR 55%‐82%) and the median proportion of estimated quantities (not weighed) was 8% (IQR 0%‐29%). Younger participants (age group 1) used more estimated household measurements instead of weighing than participants in age groups 2 and 3 (0%‐26%, 8% vs 0%‐31%, 7% and 0%‐23%, and 4%, respectively).

**Table 3.** Completion time and proportion of items already coded for the total sample (N=74) and stratified by age groups.

	Total	Age group 1: 18‐30 y	Age group 2: 31‐45 y	Age group 3: 46‐65 y
Participants, n (%)	74 (100)	41 (55.4)	15 (20.3)	18 (24.3)
Number of entered items day 1^a^	21 (15‐31)	22 (16‐31)	21 (19‐32)	21 (13‐30)
Total completion time day 1 (min)^a^	35 (19‐52)	35 (19‐45)	44 (19‐55)	36 (17‐53)
Relative completion time day 1 (min/item)^a^	1.6 (1.2‐2)	1.5 (1.1‐2)	1.5 (1.1‐2)	1.8 (1.3‐2.3)
Total completion time sample meal (min)^a^	15 (12‐18)	14 (11‐17)	17 (11‐19)	17 (13‐23)
Relative completion time sample meal (min/item)^a^	0.8 (0.7‐0.9)	0.8 (0.6‐0.9)	0.9 (0.6‐1.1)	0.9 (0.7‐1.3)
Proportion of items already coded (%)^a^	68 (55‐82)	63 (56‐79)	77 (62‐86)	71 (50‐86)
Proportion of estimated household quantities (%)^a^	8 (0‐29)	8 (0‐26)	7 (0‐31)	4 (0‐23)

a Median (IQR).

In total, 24% (18/74) of the participants reported technical issues. In 2 cases, the app crashed, but could be reopened afterward and no data was lost. In 5 cases, participants stated that they had problems finding newly added products (this process is sometimes delayed in rare cases). Further, 4 participants reported that the selection from the drop-down menu did not work properly. In the remaining cases, participants described issues rather associated with this study design than with the technical functions of NutriDiary or gave general comments. For example, 4 participants reported that a barcode presented in the sample meal could not be scanned from the screen (probably due to varying screen-brightness and screen-resolution).

Overall, 85% (63/74) of the participants were able to enter the sample meal correctly. Of the 11 participants who made mistakes when entering the sample meal, 5 were in age group 1, 2 in age group 2, and 4 in age group 3, indicating that age did not affect the accuracy of data entry. In 2 cases, food items were missing. Further, 3 participants had difficulties in editing a WDR entry and entering hypothetical leftovers. Furthermore, 6 participants entered a different amount of food than that presented in the sample meal. When participants were asked whether they would prefer NutriDiary to the traditional paper method, a total of 77% (57/74) agreed. Only 9.5% (7/74) could imagine, that it would be easier to keep a WDR with pen-and-paper than to use NutriDiary (Multimedia Appendix 2).

## Discussion

### Principal Findings

To our knowledge, an app-based WDR system with barcode scanning function for scientific studies in Germany does not exist so far. The popularity and widespread use of smartphones make NutriDiary a promising alternative to the traditional pen-and-paper approach. Different ways for entering food items (text search, barcode scanning, and free text entry) and the NutriScan function enable users to record the products they consume in a very detailed manner. The digital data output has the potential to reduce the burden for researchers also. In this study, we evaluated NutriDiary in a convenience sample of experts and laypersons and found good usability and acceptability.

The median SUS score of 75 indicated good usability of NutriDiary. The SUS score has been developed as a means to measure the overall perceived usability of a system [26,27]. However, technology-based dietary assessment instruments for scientific purposes are very specific tools that are not designed for everyday use but to generate scientifically useful data. The collection of these data is often challenging for the participants. The first statement of the SUS questionnaire is “I think that I would like to use this system frequently.” This statement is unlikely to be agreed upon by many people in the case of a WDR due to the burden on participants by the method itself. Nevertheless, the advantage of the SUS score is that it offers the possibility to compare systems used in the same context [27]. The median SUS score of 75 for NutriDiary (mean SUS score: 74, SD 15, for comparison) is comparable with SUS scores for other similar technology-based food records such as “METADIETA-web” (mean SUS score: 68, SD 15, n=26) [31], the “Eat and Track app” (mean SUS score: 69, n=15) [32] or the “Traqq app” (mean SUS score: 79, SD 15, n=22) [33].

Age has been discussed as a major limiting factor for the use of technology-based systems [29] or innovative dietary assessment instruments [31,34,35]. Consistently, the results of the backward selection procedure showed that age was identified as a potential predictor for a lower SUS score in the examined sample. Further, older participants took slightly longer to complete a WDR with NutriDiary than younger ones. Feasibility testing of the digital food record METADIETA-web by Vitale et al [31] also showed that the preference for using the digital tool instead of the traditional pen-and-paper method decreased with increasing age [31]. Older generations did not grow up with smartphones and computers and are therefore likely to be less intuitive with apps and web-based tools than younger generations. However, longer completion times could also be explained by higher accuracy in entering items and greater patience [36], which would be in line with the observation that older participants used fewer estimated household measurements instead of weighing than younger ones in our study (0%‐23%, 4% for age group 3 vs 0%‐31%, 7% and 0%‐26%, 8% for age group 2 and 1, respectively). Nevertheless, an understandable introduction to the use of the instrument might be particularly important in the group of older adults. For practical reasons, only an introductory video was sent out in this study. Our experience in training users to use the NutriDiary app shows that older people tend to ask more questions. This can only be addressed in a face-to-face conversation where it can be ensured that all relevant information is conveyed. Therefore, we recommend a personal introduction when using NutriDiary in epidemiological studies, if feasible.

The median SUS score was higher in women than in men and higher in the group of experts than in the group of laypersons. This may partly be explained by the fact that the proportion of older people was higher in men than in women and higher in the group of laypersons than in the group of experts. The group of laypersons also included students of nutritional science. Although this group certainly cannot be described as experts, the students may have some background knowledge that could have influenced the outcome. Whether this makes them more critical or less critical in their judgment remains questionable.

WDRs have the potential to provide the most accurate description of the types and amounts of the foods consumed over a specified period of time, but they are also considered to be one of the most burdensome and elaborate dietary assessment methods. When conducting a WDR, participants have to weigh and write down everything they eat and drink and always carry their kitchen scales, the record sheet, and a pen with them. This process is exhausting and time-consuming and requires a high level of cooperation from the participants [37]. The median completion time for conducting a WDR with NutriDiary was 35 minutes (1.6 min/item, Table 3) and can be rated as acceptable. When looking at the median relative completion time of the sample meal (here, the amount of the presented foods was predefined), it can be assumed that the single entering process (without weighing) takes around 0.8 minutes per item. This result suggests that weighing accounts for about half the completion time. Keeping this in mind, the single entering process is roughly comparable with the reported average completion time of other text-based DR apps, for example, with “My Meal Mate.” Here, participants needed on average 22 minutes per day to complete a record with estimated (not weighed) portion sizes [38].

Whether the NutriDiary app significantly reduces the completion time compared to the traditional pen-and-paper method is questionable, because the digital version of a WDR does not change the fact that weighing is required for this dietary assessment method. However, NutriDiary offers some advantages that can make its use more attractive than the traditional method. First, most people always carry their smartphone with them and recording “on the go” is more practical than using pen-and-paper. Presumably, this makes it less likely that participants forget their records and need to add the meal at a later time. Second, features such as the recipe book and the barcode scanner can make recording easier, as frequently consumed food combinations and recipes can be stored and retrieved later, and consumed products can be added more easily. Furthermore, integrated standard household measurements make it easier to estimate the quantity if weighing is not possible, for example, when eating out of home. The photo function allows participants to add a photo of the meal, which can help the postprocessing. To figure out whether NutriDiary is more attractive to study participants than the traditional pen-and-paper method, we added 2 more questions to the SUS questionnaire (, Multimedia Appendix 2). The result clearly showed that the vast majority would prefer using the NutriDiary app instead of a paper-based food record. To avoid errors caused by application problems using technology-based instruments, an understandable introduction and information structure are necessary to prevent frustration and enable participants to help themselves quickly if they have difficulties. For this purpose, we designed a website where all information and support materials are centralized in 1 location [24].

When using traditional DRs in epidemiological studies, the postprocessing of the DRs is very time-consuming. The data needs to be digitized and manually coded by the study staff. In this study, 68% (IQR 55%‐82%) of the food entered in NutriDiary was already coded. Furthermore, the data was already available in digital form, suggesting less costs and time in data postprocessing, compared to the pen-and-paper method. NutriDiary was designed as an app for keeping WDRs, meaning that participants are asked to weigh all the food and drinks they consume. However, as experience showed that this is not always possible, the app also offers the choice of standard household measurements (eg, teaspoon, glass, or portion) to estimate the quantity in situations where weighing is not possible. Considering the burden of weighing all foods, this selection option might tempt participants to estimate rather than weigh. However, the proportion of estimated quantities was rather low and can be considered manageable in this study.

Within the development process of NutriDiary, building an appropriate food database structure and the collection, standardization, and integration of barcode and food packaging information to enable food entry via barcode scanning was one of the most challenging tasks. The underlying database determines the users’ success in searching for food items and is essential for the functionality and accuracy of technology based, self-administered dietary assessment tools [3,39]. Therefore, we aimed to make the database as complete as possible. Due to a high number of available branded foods and the frequently changing food market, this is a major challenge [12,40-43]. According to the Food Federation Germany [44], there are more than 170,000 food products available on the German food market. Every year, about 40,000 new products are launched and just as many disappear [44]. It also happens that manufacturers change food recipes, which can also change the ingredient lists and nutritional values. These circumstances mean that the database needs to be constantly updated. To make this possible, the NutriScan function described above was developed and integrated. If the app is used regularly, the database is regularly updated with new products recorded by users.

### Strengths and Limitations

The NutriDiary evaluation study had some strengths and limitations. As NutriDiary was developed for scientific use, practicing nutritionists were also recruited as experts. Although experts are not intended users of the app, their evaluation allows for a professional view of the usability of NutriDiary. Furthermore, experts decide on the use of the assessment instrument in scientific studies, which is why their opinion on usability is of particular importance in this context. To assess user-friendliness in older age groups as well, we specifically recruited older participants. However, this study population was not representative of the general German population. Most participants were female, young, and highly educated. This limited the generalizability of our results. Nevertheless, it is important to acknowledge that participants in epidemiological studies frequently possess a higher educational status compared to the general population. In addition, participants were aware of the purpose of this study, which was to evaluate the usability and acceptability of NutriDiary, making it difficult to assess the quality and transferability of the data on the completion time and proportion of items already coded. This study aimed to assess the usability and acceptability of NutriDiary and does not contain any data on the validity of the NutriDiary app. However, providing information on the app’s validity and psychometric properties is a critical prerequisite for its effective application. Therefore, a validation study has already been initiated and will give insights into the validity and quality of the assessed nutritional data.

### Conclusion

NutriDiary is the first smartphone based WDR app with integrated barcode scanning function for scientific purposes in Germany. The evaluation by experts and laypersons indicated an acceptable completion time, good usability and acceptability on the users’ side, whereby younger experts and laypersons tended to rate the app better than older ones. Future research will give insights into the validity and feasibility of NutriDiary in different study populations.

## Supplementary material

10.2196/62776 Multimedia Appendix 1 The NutriScan process.

10.2196/62776 Multimedia Appendix 2 User rating (n=74) on participants’ preferences for NutriDiary compared to a traditional WDR (pen -and -paper method).

## Abbreviations

DONALD study: Dortmund Nutritional and Anthropometric Longitudinally Designed study
DR: dietary record
EAN: European Article Number
GTIN: Global Trade Item Number
LEBTAB: LEBensmittelTABelle, food table
SAP SE: Systems, Applications & Products in Data Processing Societas Europaea
SUS: system usability scale
WDR: weighed dietary record
