# Supplementary figures and images for: NutriDiary, a Smartphone-Based Dietary Record App: Description and Usability Evaluation
Source: JMIR Hum Factors. 2025 Feb 10;12:e62776. doi: 10.2196/62776 (PMC11833184; doi:10.2196/62776)

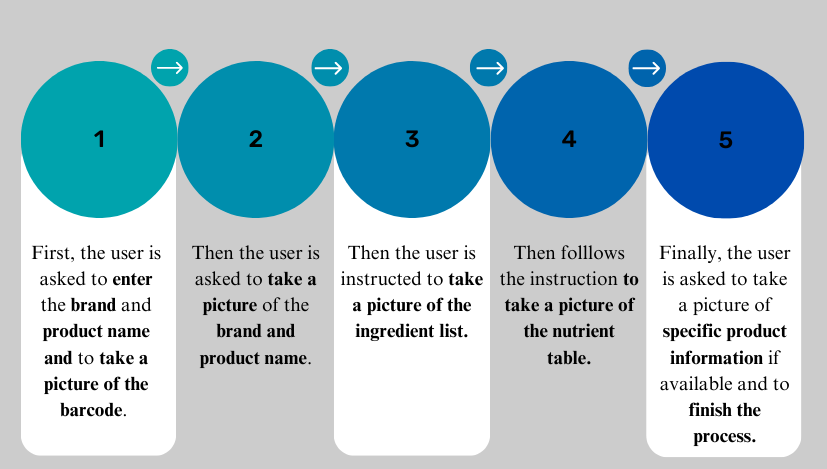

Supplement: Multimedia Appendix 1 [file humanfactors-v12-e62776-s001.png]

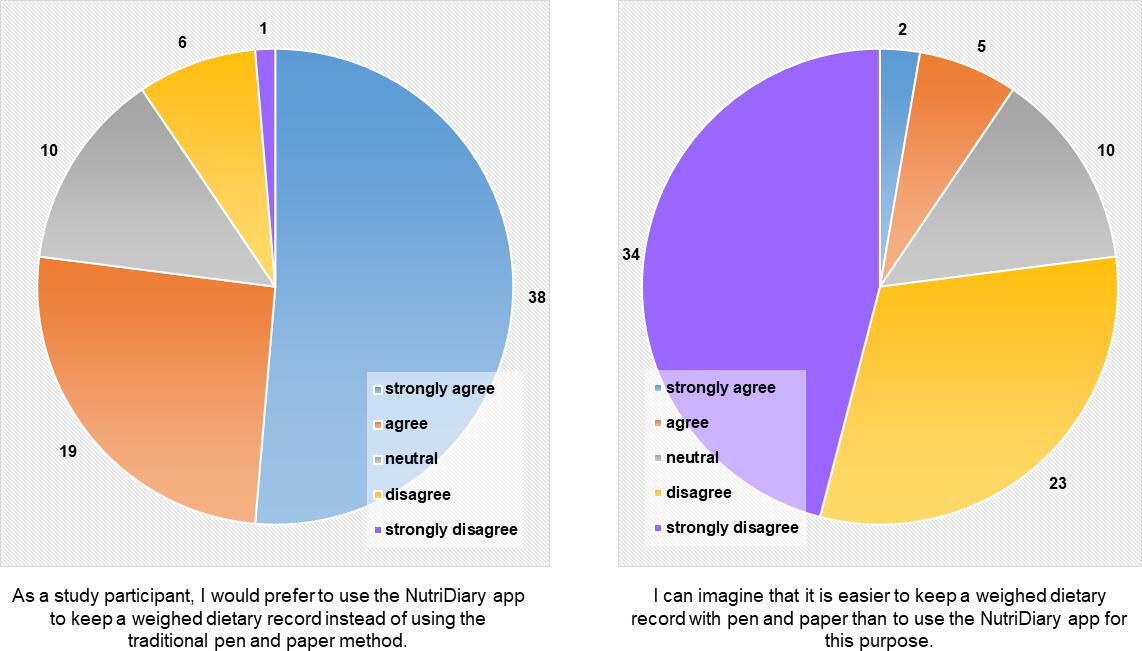

Supplement: Multimedia Appendix 2 [file humanfactors-v12-e62776-s002.jpeg]
